# Ceramides and Type 2 Diabetes: A Scoping Review of Mechanisms, Insulin Resistance, and Nutritional Interventions

**DOI:** 10.7759/cureus.108667

**Published:** 2026-05-11

**Authors:** Karin Collins, Jyotsna Chawla, Priya Krishnakumar

**Affiliations:** 1 Nutrition, Nova Southeastern University Dr. Kiran C. Patel College of Osteopathic Medicine, Davie, USA; 2 Foundational Sciences, Nova Southeastern University Dr. Kiran C. Patel College of Osteopathic Medicine, Clearwater, USA

**Keywords:** ceramides, dietary fat, insulin resistance, lipotoxicity, nutritional interventions, scoping review, sphingolipid metabolism, type 2 diabetes mellitus

## Abstract

This scoping literature review examined the relationship between ceramides and type 2 diabetes mellitus (T2DM), as well as nutrition interventions that are shown to lower circulating ceramides. Ceramides, bioactive sphingolipids implicated in lipid dysregulation, play a critical role in the development of insulin resistance in obesity-related T2DM. Since diet strongly influences ceramide synthesis, targeted nutritional interventions represent a promising strategy to counteract their impact and improve metabolic health.

A systematic search following Preferred Reporting Items for Systematic Reviews and Meta-Analyses Scoping Review (PRISMA-ScR) guidelines was conducted across PubMed, Gale Health and Medicine, Google Scholar, and EBSCO for English language articles published between 2000 and 2025. From the initial 70 records, 45 studies were selected using our systematic, rigorous selection criteria. Evidence demonstrated that high-fat diets, in particular saturated fats, elevate plasma ceramide levels, which are strongly associated with insulin resistance and T2DM. Nutrition interventions shown to lower ceramides include limiting saturated fats to less than 6% of total calories, increasing dietary fiber to ≥35 g/day, and following the Diabetes Plate guidelines.

Collectively, the evidence supports a mechanistic and associative link between ceramides and T2DM. It highlights the emerging potential of plasma ceramides as biomarkers for insulin resistance and disease risk. Continuing research into nutrition‑based ceramide modulations may inform future diabetes prevention and management strategies.

## Introduction and background

According to the International Diabetes Federation, approximately 366 million adults worldwide have diabetes, representing nearly 8% of the global population [1]. By 2030, this number is predicted to reach 552 million [2]. In 2021, 38.4 million Americans were reported to have diabetes, accounting for 11.6% of the U.S. population; two million of these individuals have type 1 diabetes, making type 2 diabetes mellitus (T2DM) the most prevalent form impacting Americans [3]. Prediabetes affects 97.6 million Americans aged 18 or older, contributing to the onset of insulin resistance and T2DM [3]. Maintaining stable blood sugar is one of the many metabolic processes that help keep the body in balance [1]. When blood sugar levels are consistently elevated due to excess glucose intake, this can contribute to the deterioration of insulin sensitivity [1].

When blood sugar rises, the hormone insulin is released to lower blood glucose levels [4]. In normal insulin function, when you eat a banana, your pancreas releases insulin, which is made and stored in beta cells [5]. The GLUT4 transporter translocates to the surface of the membrane and allows glucose to come into the cell [5]. However, when a cell is insulin-resistant, the insulin receptor site is impaired, and insulin is unable to transmit signals to initiate glucose uptake, so that glucose then accumulates in the bloodstream, raising blood sugar levels [5].

Emerging data indicate that, in addition to this, there are underlying biological processes that contribute to high blood sugar and insulin resistance [1]. Evidence shows that T2DM could result from lipotoxicity in cells, where a buildup of lipids creates a cytotoxic environment, potentially impacting the insulin signaling pathways. Ceramides are one of the lipids strongly associated with the development of these metabolic dysfunctions [1]. 

Ceramides have been shown to play a key role in elevated blood sugar levels that contribute to the development of diabetes and have implications in cardiovascular, neurodegenerative, as well as cancer, obesity, depression, and inflammation conditions [6]. Ceramides are a type of sphingolipid, a lipid molecule with a spingoid at the core as its backbone attached to a fatty acid [7]. They are essentially long-chain fatty acids that help maintain the skin’s barrier [8]. Ceramides are produced via the sphingolipid pathway, circulate in the bloodstream, and function as signaling lipids in response to stress, inflammation, or tissue damage [8]. They act as bioactive agents involved in intracellular pathways, including the production of free radicals, the release of inflammatory cytokines, the induction of apoptotic processes, and the influence on gene expression [6]. As with any nutrient, when overconsumed, excess fats can disrupt the metabolic homeostasis our body constantly strives to maintain, contributing to high blood glucose and T2DM [9].

Circulating ceramides, specifically the Cer-16 (a shorter-chain saturated fatty acid), are associated with a high risk of T2DM and cardiovascular disease [7]. People with obesity and T2DM have been shown to have elevated plasma ceramide levels, particularly C18:0, C20:0, and C24:1 [6]. The accumulation of ceramides has been found to inhibit the insulin signaling pathway in vitro [6]. Ceramides can accumulate in the pancreas, muscle, adipose tissue, brain, liver, kidney, eye, heart, and vessels, and are correlated with chronic diabetic conditions such as lipid toxicity, dyslipidemia, atherosclerotic plaque formation, and vasoreactivity [6,10]. Ceramides are transported in blood by lipoproteins such as very-low-density lipoprotein (VLDL), low-density lipoprotein (LDL), high-density lipoprotein (HDL), and chylomicrons, as well as by extracellular vesicles [11]. There are different ceramide species associated with specific tissues, such as the liver, muscle cells, adipocytes, kidneys, heart, and intestines [11]. Differences in ceramide species by tissue and circulation may be relevant to metabolic disease risk [11].

Therefore, one of the most important questions to be addressed is the association between ceramides and T2DM, and to examine how nutrition interventions can lower ceramides for T2DM management. A preliminary search of PubMed, Gale Health and Medicine, Google Scholar, and EBSCO was conducted, and no current systematic reviews covering both the above topics were identified.

Research objective

The goal of this scoping literature review was to explore the mechanistic and associative links between ceramides and T2DM, focusing on the role of ceramides in insulin resistance and then exploring nutritional interventions for lowering ceramides for the management of T2DM.

## Review

Method

Search Strategy Details

The method for this literature review consisted of gathering all clinical research and studies on ceramides related to their role in T2DM, as well as nutrition research and studies aimed at lowering plasma ceramides. The data have been compiled and organized into three main categories: 1. The role of ceramides in T2DM, 2. Dietary Contributions Elevated Ceramides, and 3. Nutrition Interventions that Lower Ceramides. The goal of the search was to identify clinical studies that measured ceramides and correlated them with insulin resistance and/or T2DM. The goal of the search for nutrition studies was to identify studies that measured or correlated the effects of foods, diets, or supplements on ceramide levels. The Preferred Reporting Items for Systematic Reviews and Meta-Analyses (PRISMA) research strategy, as shown in Figure 5, was used for sorting through the literature. Seventy research articles were initially identified across four databases: PubMed, Gale Health and Medicine, Google Scholar, and Ebsco. The keyword and key phrase search terms used to find the clinical studies on ceramides were: “nutrition AND ceramides”, “nutrition interventions for ceramides”, “ceramides AND diabetes”, “plasma ceramides AND Type 2 diabetes mellitus”, “nutrition for lowering ceramides in diabetics”, and “nutrition interventions for lowering ceramides in Type 2 diabetes.”

**Figure 1:**
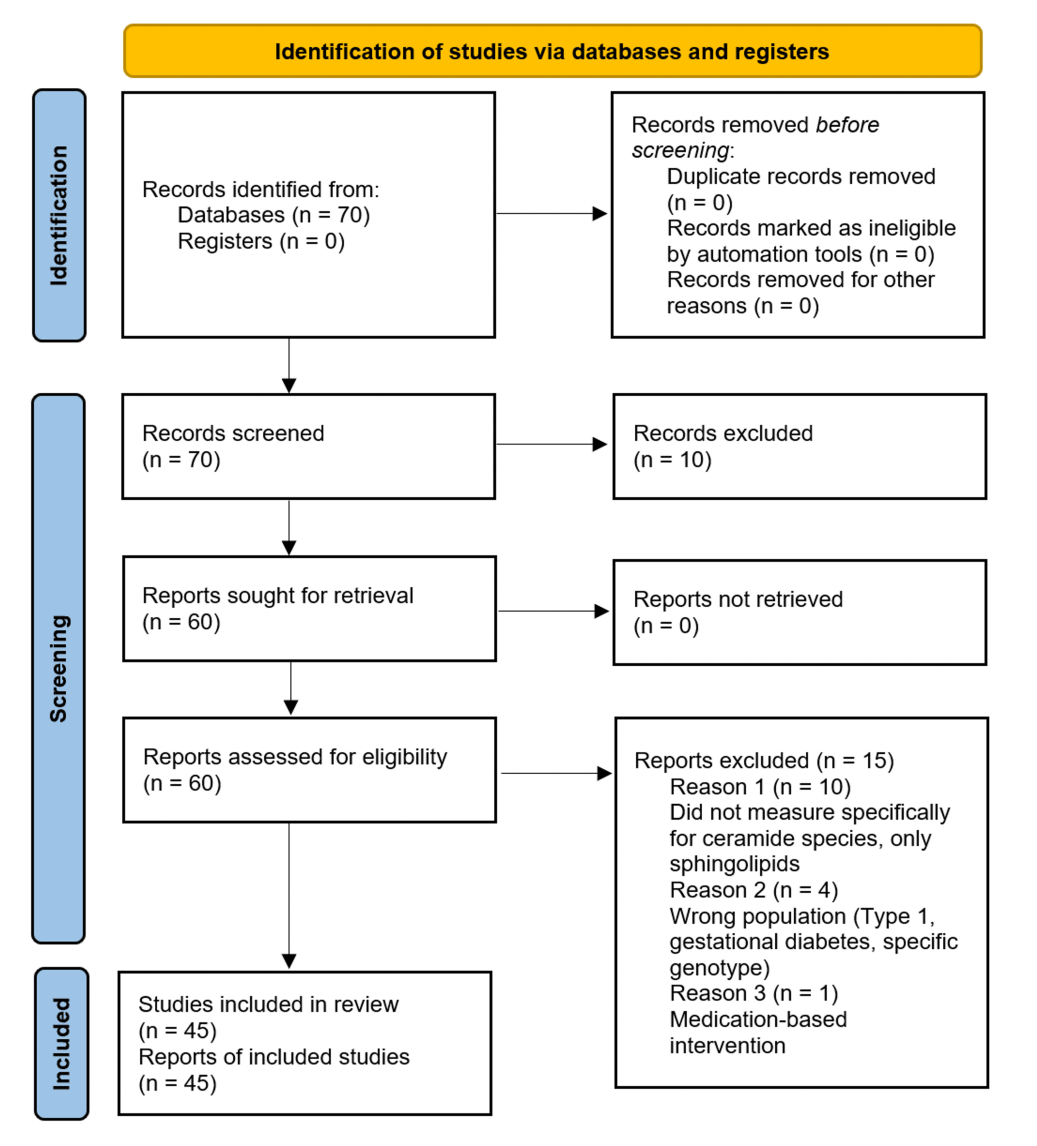
PRISMA flow chart showing identification, screening, eligibility, and inclusion of studies This PRISMA 2020 flow diagram depicts the identification, screening, eligibility assessment, and final inclusion of studies in the review. Of 70 records screened, 45 studies met the inclusion criteria and were included. PRISMA: Preferred Reporting Items for Systematic Reviews and Meta-Analyses

Inclusion/Exclusion Criteria

The inclusion criteria consisted of peer-reviewed English-language articles. The clinical studies could include adults, children, men, and/or women. The studies needed to measure ceramides and either measure the incidence of T2DM or insulin resistance, or be conducted on people who are actively diagnosed with T2DM. The dietary interventions had to involve food, diet, or nutrition supplements. The exclusion criteria consist of clinical studies done on pregnant women, people with a specific genotype, and participants who have type 1 diabetes. In the nutrition intervention studies, interventions involving medications were also excluded. After screening and reviewing the articles for eligibility, 45 studies were included in the final synthesis.

Results

Clinical Studies Linking Ceramides to T2DM

The data collected on these clinical studies show common connections between ceramides and T2DM (Table 1 [12-30]). Elevated ceramides in the plasma correlate with a higher prevalence of insulin resistance and insulin sensitivity [12-18]. Elevated ceramide levels were also strongly associated with people who have T2DM, the incidence of T2DM, and a greater risk of diabetes [12-14,16-29]. The specific ceramide species that were frequently shown to be linked to T2DM are the C16, C18, C20, C22, and C24 [13,16,19-23,28,29]. High amounts of free fatty acids in pancreatic cells are linked to beta cell death [30]. The evidence that ceramides have an associative relationship is strong because many of the studies conducted involved a large number of subjects (over 1,000), involving both men and women [15,16,21-24,28]. The methods used in these studies also involved measuring numerous specific ceramide species, allowing researchers to narrow down those linked to T2DM [13,16,19-23,28,29]. 

**Table 1:** Clinical studies that link ceramides to Type 2 diabetes mellitus LDL: low-density lipoprotein; NGT: normal glucose tolerance; IGT: impaired glucose tolerance; IL-6: interleukin 6; T2DM: type 2 diabetes mellitus; LCER: lactosylceramide; CER/Cer: ceramide; CHS: Cardiovascular Health Study; CERT2: Coronary Event Risk Test 2; ADA: American Diabetes  Association; CAD: coronary artery disease; CerRatio: ceramide ratio Cer d18:18/Cer d 18:24:0; HOMA-IR: homeostatic model assessment of insulin resistance; HDL: high-density lipoprotein; HDL-C: high-density lipoprotein cholesterol; LDL-C: low-density lipoprotein cholesterol; HbA1c: glycated hemoglobin; TNF-α: tumor necrosis factor alpha; CCL2: C-C Motif Chemokine Ligand 2; FFA: free fatty acid

Author	Study Title	Study Design	Method	Participants	Ceramide Research Findings
Boon et al., 2013 [12]	Ceramides contained in LDL are elevated in type 2 diabetes and promote inflammation and skeletal muscle insulin resistance.	Experimental and observational design: included a cross-sectional comparison of human participants, an interventional design, an in vitro cell culture experiment, and an in vivo animal experiment.	Human studies: compared lean, obese, and obese type 2 diabetic individuals, measured ceramide levels in plasma, and examined ceramide changes in obese women before and after weight loss. Mouse studies: infused LDL containing ceramide into lean mice and measured glucose uptake and insulin sensitivity. Cell Culture Studies: treated skeletal muscle cells and macrophages with LDL-ceramide to study insulin signaling and inflammation.	40 human participants and 17 mice	Elevated LDL-ceramides in the plasma correlate with insulin resistance but are not obesity dependent, meaning someone who is not obese can still have high levels of ceramides in their blood. LDL-ceramide levels were 51–72% higher in type 2 diabetics than in lean or obese, insulin-sensitive people. Hepatocytes (liver cells) from obese mice secreted 57% more ceramide than those from lean mice. Adipocytes (fat cells) and myotubes did not secrete ceramide → suggesting hepatic origin. Infusing LDL-ceramide into lean mice reduced whole-body glucose clearance by ~30%. In cultured myotubes, LDL-ceramide decreased insulin-stimulated glucose uptake by ~25%. LDL-ceramide increased TNF-α, IL-6, and *CCL2* expression and secretion.
Haus et al, 2009 [13]	Plasma ceramides are elevated in obese subjects with type 2 diabetes and correlate with the severity of insulin resistance.	Cross-sectional observational human study	Ceramide species in the plasma were measured and examined via quantitative tandem mass spectrometry in patients with obesity and diabetes and healthy, non diabetic people in the control group.	27 subjects (14 healthy non-diabetic controls, 13 type 2 diabetic patients)	Insulin sensitivity was lower in patients with type 2 diabetes than in patients in the control group. Type 2 diabetic subjects had higher concentrations of C18:0, C20:0, C24:1, and total ceramide. Insulin sensitivity was inversely correlated with C18:0, C20:0, C24:1, C24:0, and total ceramide.
Brozinick et al., 2013 [14]	Plasma sphingolipids are biomarkers of metabolic syndrome in non-human primates maintained on a Western-style diet.	Cross-sectional observational	Rhesus macaque monkeys were fed a high-fat and high-fructose diet for various periods of time and diagnosed as diabetic or pre-diabetic. Their serum ceramide levels were analyzed and correlated with insulin resistance.	27 subjects (12 non diabetic control, 10 pre-diabetic, 5 diabetic)	Plasma ceramides were found to be significantly higher in pre-diabetic and diabetic animals when compared to the control animals. The high-fat, high-fructose diet was correlated with increased insulin sensitivity.
Neeland et al., 2018 [15]	Relation of plasma ceramides to visceral adiposity, insulin resistance, and the development of type 2 diabetes mellitus: the Dallas Heart Study.	Prospective cohort study	Plasma ceramides, metabolic biomarkers, and fat depots were measured in non-diabetic participants of the Dallas Heart Study. Those participants were measured and assessed 7 years later for associations between body fat, insulin resistance, ceramide levels, and incidence of diabetes.	1557 non-diabetic participants of the Dallas Heart Study	The results showed that total cholesterol levels were associated with all types of ceramides, but high levels of triacylglycerol and low levels of HDL-cholesterol were only correlated with the saturated fatty acid chain ceramides. Insulin resistance was found to be positively correlated with saturated fatty acid ceramides and inversely correlated with polyunsaturated fatty acid ceramides. Visceral adipose tissue was found to be positively associated with saturated ceramides and inversely associated with polyunsaturated ceramides.
Berkowitz et al., 2024 [16]	Sphingolipid profiling as a biomarker of type 2 diabetes risk: evidence from the MIDUS and PREDIMED studies	Cross-sectional observational study	They measured blood sphingolipid species (ceramides and lactosylceramides) in participants from two cohorts (MIDUS and PREDIMED) and compared them to incidences of T2DM and insulin resistance.	2,961 participants	An elevated prevalence of Type 2 diabetes and insulin resistance was linked to Ceramides CER18:0 and CER22:0. However, T2DM and insulin resistance showed an inverse association with the lactosylceramides (LCER 14:0, 16:0, and 24:1).
Straczkowski et al., 2007 [17]	Increased skeletal muscle ceramide level in men at risk of developing type 2 diabetes.	Cross-sectional observational study	Muscle samples were taken from 45 men and measured for insulin sensitivity, and in those muscle samples, they quantified ceramide, sphingomyelin, sphinganine, and sphingosine levels, as well as enzyme activities (sphingomyelinase and ceramidase)	45 men	Men at risk of developing type 2 diabetes (offspring of diabetic patients and overweight/obese with NGT or IGT) had higher skeletal muscle ceramide levels compared with lean controls. Insulin sensitivity (measured by euglycaemic-hyperinsulinaemic clamp) was significantly lower in the at-risk groups vs controls (all p < 0.005).
de Mello et al., 2009 (18)	Link between plasma ceramides, inflammation, and insulin resistance: association with serum IL-6 concentration in patients with coronary heart disease.	Cross-sectional study	Serum levels of the inflammatory markers and plasma lipid metabolites were measured and compared to the amount of insulin resistance	33 patients with coronary heart disease	Serum ceramides were highly correlated with serum circulating levels of IL-6. IL-6 is a type of inflammatory marker known to contribute to insulin resistance.
Fretts et al., 2020 [19]	Plasma ceramide species are associated with diabetes risk in participants of the Strong Heart Study.	Nested case control study, parallel prospective cohort	The method used plasma samples stored from the Strong Heart Study and the Strong Heart Family Study to measure the amount and type of Sphingolipid species present, and compared it to the prevalence of diabetes among those participants.	435 American-Indian participants from the Strong Heart Study and 1902 participants from the Strong Heart Family Study.	High concentrations of three specific circulating ceramide species (consisting of behenic acid Cer-22, arachidic acid Cer-20, and stearic acid Cer-18) were correlated with a higher diabetes risk.
Wigger et al., 2017 [20]	Plasma dihydroceramides are diabetes susceptibility biomarker candidates in mice and humans.	Nested case-control/prospective cohort biomarker study design.	Participants had their fasting glucose, insulin, and lipid panel measured and then compared those measurements to their baseline plasma samples. In the mice experiment, 48 male mice were experimented on, 24 given the diabetes resistant strain, 24 given the diabetes susceptible strain, and then they were fed a high-fat diet (60% kcal from fat, source: lard - a saturated source). After 16 weeks on the high-fat diet, their plasma ceramides were measured and related to diabetes risk.	Human Cohort: 164 plasma samples. Mice Experiment: 48 mice.	Using lipidomic analysis, biomarkers were measured for risk of developing Type 2 diabetes up to 9 years before their diagnosis. Cer(d18:0/22:0), Cer(d18:0/24:0), Cer(D18:0/23:0), and Cer(d18:0/24:1) were at high levels 9 years prior to diabetes diagnosis. Cer(d18:1/22:0), Cer(d18:0/23:0), Cer(d18:1/18:0), Cer(d18:1/22:0), Cer(d18:0/16:0), Cer(d18:0/24:0), Cer(d18:0/23:0), and Cer(d18:0/24:1) were at high levels 5 years prior to diabetes diagnosis.
Fretts et al., 2021 [21]	Plasma ceramides containing saturated fatty acids are associated with the risk of type 2 diabetes.	Prospective cohort study, observational, and longitudinal.	22 plasma sphingolipid species were measured in the plasma samples among the CHS participants who did not at that time have diabetes. Incidence of diabetes among those participants was determined based on their current fasting glucose tests.	3,645 CHS participants	In elderly adults, elevated levels of Cer-22, Cer-20, Cer-18, and Cer-16 were correlated with a greater risk of diabetes.
Hilvo et al., 2018 [22]	Ceramide stearic to palmitic acid ratio predicts incident diabetes	Prospective cohort study, interventional trial.	Ceramide species Cer(d18:1/16:0), Cer(d18:1/18:0), Cer(d18:1/24:0), and Cer(d18:1/24:1) were measured from plasma samples and correlated with incidences of diabetes.	9,302 total non-initially diabetic participants were included in the study.	Stearic acid ceramide (18:0) shows the strongest association with incident diabetes, and the ceramide ratio of Cer(d18:1/18:0)/Cer(d18:1/16:0) was shown to predict incidences of diabetes.
Chen et al., 2020 [23]	Serum sphingolipids and incident diabetes in a US population with high diabetes burden: the Hispanic Community Health Study/Study of Latinos (HCHS/SOL).	Prospective population-based study.	Participants were interviewed and clinically assessed for their fasting blood glucose at baseline and then re-examined years later to determine the onset of diabetes outcomes. Their fasting glucose was measured, and they were given a sphingolipid score with their risk of diabetes.	2010 participants (from Health Study and Study of Latinos), aged 18-74 years old, and without diabetes at baseline.	Cer-16 and Cer-18 were shown to be associated with a greater risk of diabetes in participants from the Latino Health Study (aged 34-41).
Yun et al., 2020 [24]	Associations among circulating sphingolipids, β-cell function, and risk of developing type 2 diabetes: A population-based cohort study in China.	Prospective cohort study.	Ceramides, fasting glucose, HbA1c, insulin, total cholesterol, LDL-C, HDL-C, and triglycerides were measured in blood samples and correlated with incident Type 2 diabetes and Type 2 diabetes risk.	1,974 Chinese men and women.	3 monounsaturated ceramides, 7 sphingomyelins, and 1 hexosylceramide were shown to be positively correlated with incident Type 2 diabetes in the Chinese population.
Wilmott et al., 2019 [25]	Analysis of sphingolipid composition in human vitreous and changes associated with diabetic retinopathy.	Observational study.	This study analyzed the sphingolipid composition of human vitreous samples from post-mortem human eyes of non-diabetic (control) and type 2 diabetic cadavers.	13 human vitreous samples were used.	Total ceramide levels were significantly higher in T2DM samples.
Leiherer et al., 2022 [26]	The ceramide- and phosphatidylcholine- based Coronary Event Risk Test 2 (CERT2) and cardiovascular mortality in men and women with type 2 diabetes.	Prospective cohort study.	The CERT2 risk score was taken at baseline and then compared with mortality rates when following participants after 7-8 years.	401 participants with type 2 diabetes mellitus	The CERT2 score strongly predicted the occurrence of cardiovascular death rates in male patients with yype 2 diabetes mellitus.
Leiherer et al., 2024 [27]	Ceramides predict the development of type 2 diabetes.	Prospective cohort study.	Ceramide ratio was analyzed in 894 patients. Patients were followed for up to 16 years, tracking the incidence of T2DM, using the ADA criteria to diagnose T2DM.	894 Caucasian patients referred for CAD evaluation.	Patients with type 2 diabetes mellitus had higher CerRatios than the non-diabetic patients. During the follow-up, 11% of the non-diabetic patients were newly diagnosed with T2DM, and the CerRatio was a strong predictor of T2DM incidence.
Dugani et al., 2021 [28]	Association of plasma ceramides with prevalent and incident type 2 diabetes mellitus in middle-aged and older adults.	Prospective population-based cohort.	The association of ceramides with the prevalence of type 2 diabetes mellitus was analyzed at baseline, and the incidence of T2DM was examined during a follow-up 6.2 years later.	1,423 adults	Ceramides (C16:0, C18:0, C18:0/C16:0 ratio, C18:0/C24:0 ratio) were positively correlated with the prevalence of type 2 diabetes mellitus. Ceramides (C18:0, C18:0/C16:0 ratio) were associated with the incidence of T2DM.
Lopez et al., 2013 [29]	Plasma ceramides are elevated in female children and adolescents with type 2 diabetes.	Cross-sectional study.	Fasting blood samples were collected, and plasma ceramide subspecies were measured using quantitative tandem mass spectrometry. Measures of adiponectin, HOMA-IR, BMI z-score, triglycerides, and fasting glucose were also collected to assess correlations with ceramide levels.	28 participants	Plasma ceramides were significantly more elevated in female children with type 2 diabetes than in the non-diabetic control group. Suggesting that ceramides play a role in early disease development. Participants with type 2 diabetes had higher levels of ceramides C22:0, C20:0, C18:0, and C24:1 and increased HOMA-IR, BMI, triglyceride, and fasting blood glucose.
Lupi et al., 2002 [30]	Prolonged exposure to free fatty acids has cytostatic and pro-apoptotic effects on human pancreatic islets: evidence that beta-cell death is caspase-mediated, partially dependent on the ceramide pathway, and Bcl-2 regulated.	In vitro experimental study.	Human pancreatic islets were isolated from 18 non-obese organ donors and cultured with free fatty acids for 48 hours and then tested for their insulin secretion function, glucose utilization, and cell survival under lipotoxic conditions.	18 non-obese organ donors.	The pancreatic islets isolated for 48 hours with 2.0 mmol/free fatty acids showed a great reduction of glucose metabolism with a decrease in glucose oxidation down to 40% and a decrease in glucose utilization down to 50%. In the control pancreatic islets, after 48 hours of incubation with free fatty acids, there was a higher trend of insulin release. There was also more cell death happening among the pancreatic islets exposed for 48 hours to 2.0 mmol/FFA than in the control islets.

Clinical Studies on Dietary Implications of Elevated Ceramides

Data were collected from clinical studies showing dietary intakes that are correlated with elevated ceramides (Table 2 [14,21,31-35]). The clinical findings showed that saturated fatty acids are positively associated with high plasma ceramides [21,31]. Many studies also found that a high dietary fat intake is associated with increased total ceramide levels [14,32,33]. Other dietary factors contributing to elevated plasma ceramides include the intake of nuts, sugar-sweetened beverages, high-fructose diets, and high-calorie/overfeeding [14,32-35]. The high-calorie diets showed that dietary interventions of a caloric surplus of 1,000 - 1,200 calories over necessary energy needs are correlated with increased total ceramides outcomes as early as three to four weeks following those interventions. High dietary fat intake in general is associated with elevated ceramides, but saturated fats have a greater effect on ceramide synthesis. The high-fat dietary interventions used dietary fat protocols of 36%, 44%, and 45% of total intake, and in each of those studies, those diets led to elevated ceramide levels [14,32,33]. That data suggests that consuming as little as 36% of total intake from dietary fat could contribute to elevated ceramide levels. This provides further evidence that intake of 35% or less dietary fat is associated with lower total ceramide levels and may help prevent ceramide accumulation.

**Table 2:** Clinical Data on the Dietary Contributions to Elevated Ceramides CHS: Cardiovascular Health Study; Cer: ceramide; FFQ: Food Frequency Questionnaire; GM: GM3 ganglioside; SM: sphingomyelin

Author	Study	Nutrient Category	Study Design	Method	Participants	Findings
Fretts et al., 2021 [21]	Plasma ceramides containing saturated fatty acids are associated with the risk of type 2 diabetes.	Saturated fatty acids	Prospective cohort study, observational, and longitudinal.	22 plasma sphingolipid species were measured in the plasma samples among the CHS participants who did not at that time have diabetes. Incidence of diabetes among those participants was determined based on their current fasting glucose tests.	3,645 CHS participants	In elderly adults, elevated levels of Cer-22, Cer-20, Cer-18, and Cer-16 were correlated with a greater risk of diabetes.
Luukkonen et al., 2018 [31]	Saturated fat is more metabolically harmful for the human liver than unsaturated fat or simple sugars.	Saturated fat vs unsaturated fat vs simple sugars.	Randomized control trial.	In a 3-week parallel study, participants were assigned a 1,000 kcal/day overfeeding high in simple sugars, saturated fat, or unsaturated fat.	38 overweight adults	In the saturated fat overfeeding group, total ceramide, ceramides Cer(d18:0/24:0), Cer(d18:1/24:0), Cer(d18:2/23:0), and Cer(d18:1/24:1) increased. No changes in ceramides were found in the unsaturated fat or carbohydrate overfeeding groups.
Brozinick et al., 2013 [14]	Plasma sphingolipids are biomarkers of metabolic syndrome in non-human primates maintained on a Western-style diet.	High-fat, high-fructose diet.	Cross-sectional observational.	Rhesus macaque monkeys were fed a high-fat and high-fructose diet for various periods of time and diagnosed as diabetic or prediabetic. Their serum ceramide levels were analyzed and correlated with insulin resistance.	27 subjects (12 non diabetic control, 10 prediabetic, 5 diabetic)	Plasma ceramides were found to be much higher in prediabetic and diabetic animals when compared to the control animals. The high-fat, high-fructose diet was correlated with increased insulin sensitivity.
Covington et al., 2017 [32]	Intramyocellular lipid droplet size rather than total lipid content is related to insulin sensitivity after 8 weeks of overfeeding.	High-calorie and high-fat intake.	Prospective cohort	8-week period of overfeeding: 140% of kcal to maintain body weight, 44% of kcal from fat	29 adult men	Total ceramides and ceramides Cer(d18:1/18:0), Cer(d18:1/22:0), Cer(d18:1/16:0), Cer(d18:1/24:1), Cer(d18:1/20:0), Cer(d18:1/18:1), and Cer(d18:1/24:0) were elevated after the overfeeding period when compared to the baseline measurements.
Heilbronn et al., 2013 [33]	The effect of short-term overfeeding on serum lipids in healthy humans.	High-calorie and high-fat intake.	Prospective cohort	This study consisted of a 4-week period of consuming 1,250 calories more than at baseline, 45% of those calories coming from fat.	40 healthy adults	Total ceramide levels increased when measured after the overfeeding period. Cer(d18:1/24:0), GM3(d18:1/22:0, Cer(d18:1/22:0), GM3(d18:1/24:0), Cer(d18:0/24:0), Cer(d18:0/22:0), Cer(d18:1/24:0), and Cer(d18:1/22:0) increased post overfeeding. Cer(d18:1/24:1), Cer(d18:0/18:0), Cer(d18:1/24:1), Cer(d18:1/18:0), GM3(d18:1/24:1), and GM3(18:1/18:0) reduced post overfeeding.
Walker et al., 2020 [34]	The Framingham Offspring Study	Sugar-sweetened beverages.	Observational cohort.	Sugar-sweetened beverage intake assessed from Harvard FFQ.	124 participants from the Framingham Offspring Study	Intake of sugar-sweetened beverages was positively correlated with Cer(d18:1/22:0) and Cer(d18:1/16:0) and positively correlated with Cer(d18:1/24:0) only in participants with prediabetes or diabetes.
Malik et al., 2019 [35]	Identification of plasma lipid metabolites associated with nut consumption in US men and women.	Nuts.	Cross-sectional.	Frequency of nut intake assessed from FFQs.	1,099 subjects from the Health Professionals Follow-up Study, Nurses’ Health Study, and Nurses’ Health Study II.	The intake of nuts was positively associated with Cer(d18:1/24:0), SM(d18:1/24:0), and SM(d18:1/22:0) and negatively correlated with SM(d18:1/18:0).

Nutrition Intervention Studies to Lower Ceramides

The nutrition intervention studies found in the research showed many promising nutrition interventions for lowering ceramides (Table 3 [36-56]). In some cases, the Mediterranean diet was associated with lower ceramide levels [36-39]. In cases with elevated or unchanged ceramide levels, the disruptive effects of ceramides on lipid metabolism were inhibited [37]. Diets higher in plant foods (including whole grains, fruits, and vegetables) were associated with lower ceramide levels [36,40-42]. The Nordic diet, which consists of fruits, vegetables, fish, low-fat dairy, low-fat meat, whole grains, and vegetable oils, was shown to decrease insulin resistance and plasma ceramide concentrations [43]. When a vegetarian diet was compared with a meat-based diet, the vegetarian group showed lower levels of ceramides and a lower variety of ceramide species [44].

**Table 3:** Nutrition Interventions for Lowering Plasma Ceramides Cer: ceramide; CERT2: Coronary Event Risk Test 2; CVD: cardiovascular disease; FRUVED: fruit and vegetable; GlcCer/LacCer: glycosylated ceramides; EVOO: extra-virgin olive oil; PUFA: polyunsaturated fatty acid; SFA: saturated fatty acid; FFQ: Food Frequency Questionnaire; HFD: high-fat diet; HIF: hypoxia-inducible factors

Author	Study Title	Nutrition Category	Study design	Method	Participants	Nutrition & Ceramides Research Findings
Walker et al., 2020 [36]	The Framingham Offspring Study.	Dietary guidelines adherence and the Mediterranean diet.	Observational cohort.	Plasma ceramides were measured and evaluated for cross-sectional associations of the Mediterranean-style Diet Score (MDS) and Dietary Guidelines Adherence Index (DGAI) using multivariable linear regression models.	2157 participants of the Framingham Offspring Study.	Both diets showed that the higher the diet quality scores, the lower the levels of some of the shorter-chain ceramides. People with high scores in the MDS showed a higher ratio of very long chain ceramides to long chain ceramide (C24:0/C16:0).
Dainode et al., 2024 [37]	Mediterranean diet effects on vascular health and serum levels of adipokines and ceramides.	Mediterranean diet.	Randomized clinical trial.	Participants were randomized to a Mediterranean diet group and a low-fat diet group for 12 months, with assessments of adherence during follow-up visits every three months. Serum lipids were measured at the 6-month and 12-month follow-up visits.	101 participants	Participants in the Mediterranean diet group had lower levels of Cer-24 and higher levels of Cer-22 and Cer-24/Cer-16 ratio at the 6-month follow-up. At the 12-month follow-up, that group showed significantly lower levels of C24:0 and C18, and higher levels of Cer-24/Cer-16 ratio.
Lindqvist et al., 2021 [38]	A randomized controlled dietary intervention improved the serum lipid signature towards a less atherogenic profile in patients with rheumatoid arthritis.	Mediterranean diet.	Cross-over randomized control trial.	Participants were randomly assigned a Mediterranean diet intervention or a Western diet (control) for 10 weeks with a 2–5 month washout period.	50 adults with rheumatoid arthritis	CERT2 risk score and total serum ceramides increased during the control diet feeding period but did not decrease after the Mediterranean diet intervention.
Wang et al., 2017 [39]	Plasma ceramides, Mediterranean diet, and incident cardiovascular disease in the PREDIMED trial.	Mediterranean diet.	Observational cohort study.	In the PREDIMED trial, participants were randomly assigned to a Mediterranean diet supplemented with nuts, a Mediterranean diet supplemented with extra virgin olive oil, or a control diet. This study measured serum ceramide concentrations from participants in each of those groups using liquid chromatography.	980 subjects from the PREDIMED trial, 25% with CVD.	Ceramide concentration changes during the first year of follow-up did not differ significantly between the Mediterranean diet group and the control group.
Mathews et al., 2017 [40]	The FRUVEDOMIC pilot study: an 8-week dietary intervention increasing fruits/vegetables in young adults reduced serum ceramide levels.	Fiber and phytochemicals.	Pilot intervention study, randomized trial.	Participants were randomized into three dietary intervention groups for 8 weeks, all of which included the daily consumption of 2.5-3 cups of vegetables and 2 cups of fruit. The three groups were FRUVED, FRUVED with low refined carbohydrates, and FRUVED with low fat. Serum ceramide levels were measured before and after their intervention.	36 overweight young adults	Total ceramide levels (Cer(d18:1/22:0), Cer(d18:1/24:1), Cer(d18:1/26:0) significantly decreased in the FRUVED plus low refined carbohydrates group and the FRUVED plus low fat group at 5 weeks into the intervention. Those groups also saw a reduction in the levels of ceramides GlcCer(d18:1/22:0), GlcCer(d18:1/24:0), GlcCer(d18:1/24:1), LacCer(d18:1/22:0), and LacCer(d18:1/24:0). All the groups showed increased levels of Cer(d18:1/16:0) at the 8-week point compared to baseline.
Lankinen et al., 2011 [41]	Whole Grain products, fish, and bilberries alter glucose and lipid metabolism in a randomized, controlled trial: the Sysdimet study.	Whole grains, fish, bilberries.	Randomized controlled trial.	This study consisted of a 12-week intervention where participants were randomized into one of three dietary intervention groups. The first group was the healthy diet group, which consisted of consuming fatty fish, whole grains, bilberries, and low-insulin-response grains. The second group was the whole-grain-enriched diet, and the third group was the control group, which consumed refined wheat breads. Plasma lipids and oral glucose tolerance were measured before and after the dietary intervention.	106 adults with metabolic syndrome.	No change in plasma ceramides was found.
Drazba et al., 2019 [42]	Associations of adiposity and diet quality with serum ceramides in middle-aged adults with cardiovascular risk factors.	Diet quality.	Cross-sectional observational study.	Dietary intake data were collected using 24-hour recalls and phone interviews. Plasma ceramides were also measured and converted into a ceramide risk score. The food and nutrient intake data were correlated with their ceramide risk scores as well as compared with adherence to the dietary guidelines for Americans.	96 middle-aged adults (45 to 64 years old).	High consumption of vegetables and whole grains and low consumption of saturated fats and added sugar were correlated with lower c22:0 values. Saturated fat consumption was strongly and positively correlated with c22:0.
Lankinen et al., 2015 [43]	A healthy Nordic diet alters the plasma lipidomic profile in adults with features of metabolic syndrome in a multicenter randomized dietary intervention.	Healthy Nordic diet.	Randomized control trial	Participants were randomized to either a healthy Nordic diet or a control diet intervention for 18-24 weeks. The healthy Nordic diet consisted of fruits, vegetables, berries, whole grains, fish, low-fat meat, low-fat dairy, and vegetable oils. The control diet resembled an average Nordic diet consisting of regular milk, high-fat dairy, low-fiber cereal products, and small amounts of vegetables and fruits.	156 adults with metabolic syndrome.	Plasma ceramides correlated positively with concentrations of total cholesterol. In the healthy Nordic diet group, cer(d18:1/23:0), cer(d18:1/24:0), and cer(d18:1/22:0) were reduced after 8 weeks into the intervention, and then by 18 and 24 weeks, the differences between the two groups leveled off. insulin resistance-inducing ceramides were decreased in the healthy Nordic diet group.
Djekic et al., 2020 [44]	Effects of a lacto-ovo-vegetarian diet on the plasma lipidome and its association with atherosclerotic burden in patients with coronary artery disease.	Lacto-ovo-vegetarian diet.	Randomized, open-label, crossover study.	Subjects were randomized into either an isocaloric diet with meat or a lacto-ovo-vegetarian diet for 4 weeks, with a 4-week washout period in between.	27 CAD patients	Ceramides were lower in the vegetarian diet than in the meat diet. Cer(d18:1/22:0) and cer(d18:1/16:0), lac cer(d18:1/16:0), and hex cer(d18:1/22:0) reduced in the vegetarian diet from baseline.
Tuccinardi et al., 2019 [45]	Mechanisms underlying the cardiometabolic protective effect of walnut consumption in obese people: a cross-over, randomized, double-blind, controlled inpatient physiology study.	Walnuts.	Cross-over, double-blinded, randomized controlled trial.	Participants consumed 48 g of walnuts or a placebo daily for 5 days with a 1-month washout period.	10 obese adults.	The consumption of 48 g of walnuts daily was inversely correlated with total ceramides.
Tuccinardi et al., 2021 [46]	An extra virgin olive oil–enriched chocolate spread positively modulates insulin-resistance markers compared with a palm oil–enriched one in healthy young adults.	Palm oil vs. EVOO.	Cross-over, double-blinded, randomized controlled trial.	In a dietary intervention, participants were randomly assigned to consume chocolate spread enriched with either EVOO or palm oil daily for 2 weeks.	20 healthy adults.	Cer(d18:1/16:0)/cer(d18:1/22:0)+cer(d18:1/24:0) ratio, cer(d18:1/16:0), and sm(d18:1/18:0) were lower in the EVOO group and higher in the palm oil group.
Rosqvist et al., 2019 [47]	Overeating saturated fat promotes fatty liver and ceramides compared with polyunsaturated fat: a randomized trial.	PUFA vs SFA.	Randomized trial.	In an 8-week parallel study, participants were overfed muffins enriched with palm oil (SFA) or sunflower oil (PUFAs), followed by a 4-week period of caloric restriction.	61 overweight adults	The group that was overfed the muffins with saturated fatty acids had an increase in most of the sphingolipids. The group that consumed the muffins with the polyunsaturated fats was correlated with a reduction in all sphingolipids except cer(d18:0/24:0) and cer(d18:1/24:0).
Kien et al., 2013 [48]	A lipidomics analysis of the relationship between dietary fatty acid composition and insulin sensitivity in young adults.	Palmitate vs oleic acid.	Cross-over, randomized controlled trial.	3-week dietary intervention where participants were randomly assigned to either a high or low palmitate diet or a high-oleic acid diet, separated by a 1-week washout.	18 healthy adults.	Plasma ceramides were more elevated in the high palmitate diet group than in the high oleic acid diet group.
Seah et al., 2019 [49]	Dietary fat and protein intake in relation to plasma sphingolipids as determined by a large-scale lipidomic analysis.	Dietary fat intake and protein intake.	Cross-sectional.	Intake of dietary fat and protein measured using food frequency questionnaires (FF Qs).	2,860 participants from the Singapore prospective study program.	Ceramides were positively correlated with saturated fatty acid intake. Cer(d18:1/16:0), hex cer(d18:1/16:0), cer(d18:1/18:0), and hex Cer(d18:1/18:0) were negatively correlated with polyunsaturated intake. There was no association between plasma sphingolipids and monounsaturated fatty acids. Protein intake was negatively correlated with the majority of the sphingolipid species, and positively correlated with species with a 16:1 backbone.
Meikle et al., 2015 [50]	Postprandial plasma phospholipids in men are influenced by the source of dietary fat.	Dairy fat vs soy oil.	Cross-over, randomized controlled trial.	Participants are randomly assigned breakfast meals consisting of soy oil or dairy fat.	16 healthy adult men.	Total ceramide and sphingomyelin levels increased following the dairy fat meal and decreased following the soy oil meal.
Malik et al., 2019 [35]	Identification of plasma lipid metabolites associated with nut consumption in US men and women.	Nuts.	Cross-sectional.	Frequency of nut intake assessed from FFQs.	1,099 subjects from the Health Professionals Follow-Up Study, Nurses’ Health Study, and Nurses’ Health Study Ii.	The intake of nuts was positively associated with cer(d18:1/24:0), sm(d18:1/24:0), and sm(d18:1/22:0) and negatively correlated with sm(d18:1/18:0).
Fumeron et al., 2020 [51]	Dairy consumption is associated with lower plasma dihydroceramides in women from the D.E.S.I.R. cohort.	Dairy.	Observational.	Cheese and non-cheese dairy consumption assessed with FFQs.	105 healthy adults	Non-cheese dairy intake was inversely correlated with total dihydroceramide, cer(d18:0/24:1), cer(d18:0/24:0), cer(d18:0/16:0), cer(d18:0/22:0), cer(d18:1/26:1), cer(d18:0/23:0), and cer(d18:1/26:0).
Helge et al., 2011 [52]	Muscle ceramide content is similar after 3 weeks' consumption of a fat or carbohydrate diet in a crossover design in patients with type 2 diabetes.	Fat vs carb-rich diet.	Randomized control trial.	Each participant consumed a fat-rich diet and a carbohydrate-rich diet in two separate 3-week periods.	11 type 2 diabetic sedentary male patients.	After 3 weeks on either the fat-rich or carbohydrate-rich diet, muscle ceramide levels were not significantly different between the two diet periods.
Lankinen et al., 2009 [53]	Fatty fish intake decreases lipids related to inflammation and insulin signaling—a lipidomics approach.	Fatty fish vs. lean fish.	Randomized control trial.	A parallel study where participants consumed a diet with fatty fish, lean fish, or a control diet for 8 weeks.	33 subjects with recent acute myocardial infarction or unstable ischemic attack.	Total plasma ceramides reduced from the baseline in the group that consumed fatty fish.
Ottestad et al., 2012 [54]	Fish oil supplementation alters the plasma lipidomic profile and increases long-chain PUFAs of phospholipids and triglycerides in healthy subjects.	Fish oil.	Randomized controlled trial, double-blinded.	A parallel study in which participants took 8 g of fish oil or sunflower oil daily for 7 weeks.	33 healthy adults.	In the fish oil group, the very long-chain sphingolipid species were significantly elevated. There was no change in ceramide levels.
Xia et al., 2022 [55]	Berberine reduces hepatic ceramide levels to improve insulin resistance in HFD-fed mice by inhibiting HIF-2α.	Berberine supplement.	Animal intervention experimental study.	Mice were fed an HFD to induce hepatic hypoxia accumulation and elevate ceramide levels. Lipidomic analyses were performed on liver and/or plasma samples to quantify ceramide species and changes after intervention. Berberine treatment was administered to test whether it could reduce hypoxia, lower ceramide production, and improve insulin resistance in these HFD mice.	13 mice.	Elevated HIF-2 expression, insulin resistance, and disorder lipid metabolism in the liver of HFD-induced mice. Berberine decreases HIF-2 expression and the ceramides related to insulin resistance. Berberine promotes glycogen synthesis via the PP2A-AKT-GSK3 β signaling pathway. Berberine alleviates glucose and lipid metabolism by inhibiting HIF-2.
Deng et al., 2020 [56]	Dietary inulin decreases circulating ceramides by suppressing neutral sphingomyelinase expression and activity in mice.	Fiber supplement (inulin).	Animal intervention experimental study.	In this animal study, mice were fed an atherogenic diet and supplemented with inulin or cellulose (control group). Plasma ceramides were measured at 10 days and/or 12 weeks after the intervention.	36-40 mice.	Ceramide ratio C16:0/C24:0 decreased more in the inulin-fed mice than in the control group.

In studies that compared saturated fat intake versus unsaturated fat intake, saturated fats were consistently associated with increasing ceramide levels, whereas unsaturated fat intakes either had an inverse association with ceramide levels or no association [45-50]. On the other hand, one study showed that non-cheese dairy consumption was associated with lower plasma ceramides [51]. One study found an association between fatty fish intake and lower total ceramide levels, whereas another trial found that fish oil supplementation did not affect ceramide levels. When a high-fat diet was compared to a high-carbohydrate diet, there was no significant difference between the two diet groups’ ceramide levels [52]. These studies also showed that high-fat intake is problematic for ceramide levels, but that saturated fat intake has a stronger effect on increasing ceramide levels [53,54]. Supplements found to be beneficial for lowering ceramides include berberine and inulin [55,56].

Diet plays a significant role in the prevention, management, and treatment of T2DM, as it is often diet-induced [57]. When putting together a nutrition intervention for diabetes, it is important to address dietary factors that contribute to its development. This nutrition intervention is designed as a list of potential strategies to influence ceramide levels and maintain stable blood sugar levels. Numerous nutrition interventions have been studied for their effects on lowering plasma ceramides.

Nutrition Intervention

Based on clinical data and research findings, the nutrition interventions associated with lowering ceramides for the management of T2DM include: 1. Total Dietary Fat Intake: 20-35% of total calories, with less than 6% of total calories from saturated fat. 2. High Fiber Diet: Consume at least 35 grams of fiber and at least three cups of vegetables. 3. Diabetes Plate Pattern: To manage the glycemic load of meals, adhere to the Diabetes Plate meal pattern, which consists of ½ of the plate coming from non-starchy vegetables, ¼ of the plate consisting of a lean protein, and ¼ of the plate consisting of high-fiber carbohydrates (starchy vegetables, whole grains, fruit, legumes) [3].

Recommendation for Dietary Fats

The dietary factor consistently correlated with elevated plasma ceramides is excess intake of dietary fats, so to address lowering ceramide levels, we want to review the ideal amount of fat humans need to function. According to the World Health Organization and the Dietary Reference Intakes, the dietary guidelines for total dietary fat are 20-35% of total calories [58]. The minimum of 20% accounts for meeting our requirements for fat-soluble vitamins, essential fatty acids, and total energy [58]. That minimum requirement also helps prevent atherogenic dyslipidemia, a condition characterized by low levels of high-density lipoprotein cholesterol (HDL-C) and high triglyceride-rich lipoproteins, which can occur when consuming too little fat and too much carbohydrate, and which increases our risk of coronary heart disease [58]. According to the Dietary Guidelines for Americans 2020 - 2025, depending on activity level, most women require 1600 - 2,400, and most men require 2,400 - 3,000 calories per day [58]. That means that 20-35% of dietary fat will be anywhere from 35g to 90g for women and 53g to 117g for men. Knowing the range of fats to stay within can help us achieve the right amount of dietary fat intake to prevent under- or overconsumption. Excessive dietary fat intake over time is frequently correlated with the development of chronic metabolic conditions [58].

Saturated vs. Unsaturated Fats

Evidence suggests that there is no minimum requirement for saturated fats; there are only recommendations to limit them. Saturated fats come from animal foods, which contain many important vitamins we need, but it is recommended to limit them due to their atherogenic and diabetogenic effects [59]. The World Health Organization, the United States Department of Health and Human Services, and the United States Department of Agriculture recommend limiting saturated fat intake to less than 10% of total energy intake [59]. On a 2,000-calorie diet, 10% would be 200 calories and about 22 grams. The American Heart Association and American College of Cardiology both recommend a saturated fat intake of 5-6% of total energy [59]. 5-6% of a 2,000-calorie diet would be around 10-12 grams.

High-Fiber Diet

A high-fiber diet or a diet high in plant foods has been shown to lower ceramide levels [31,43-45]. A high fiber diet is also protective against all-cause mortality [60]. Consuming high amounts of fiber can also help stabilize blood sugar levels, which is important for managing diabetes [60]. Carbohydrates that are bound with fiber, such as whole grains, starchy vegetables, fruit, nuts, and seeds, are digested more slowly than carbohydrates without fiber, such as refined carbohydrate sources (white bread, white rice, pastries, white pasta) [60]. The recommendation of at least 35 grams per day of fiber is based on clinical evidence that shows ceramide levels decrease with that amount of plant food.

Nutrition Monitoring

The literature and research provide strong evidence that plasma ceramides play a role in the development of T2DM. The clinical implications of this research could help shape the way T2DM is diagnosed and treated. In the diagnostic process, measuring plasma ceramides could help assess diabetes risk and insulin resistance. In monitoring diabetes treatment, measuring ceramide levels could help people determine whether their dietary changes are contributing to improved diabetes outcomes. Another potential way to assess ceramide levels is to examine LDLs, since some ceramide species are found within LDL particles [12]. Conducting 24-hour recalls during patients' nutrition counseling sessions can help assess adherence to diabetes nutrition interventions.

Discussion

The current body of literature demonstrates a strong and consistent association between elevated plasma ceramides and T2DM (T2DM). Numerous large-scale clinical studies, many involving cohorts exceeding 1,000 participants and evaluating multiple ceramide species, report significantly higher circulating ceramide concentrations in individuals with insulin resistance and T2DM [15,16,21-24,28]. These findings support the growing recognition of ceramides as clinically relevant lipid biomarkers associated with metabolic dysfunction.

Nutritional research further strengthens this association by highlighting the role of dietary fat quality in ceramide metabolism. Across intervention and observational studies, diets high in saturated fatty acids are consistently associated with elevated circulating ceramide levels, whereas replacing saturated fats with unsaturated fats, particularly polyunsaturated fatty acids, is associated with reductions in ceramide levels. These findings suggest that macronutrient composition, rather than total fat intake alone, may play a critical role in modulating sphingolipid metabolism and metabolic risk.

A scoping review by Nicholson et al., “The Lard Works in Mysterious Ways: Ceramides in Nutrition-Linked Chronic Disease,” identified 15 clinical studies linking dietary ceramides to chronic diseases, including T2DM, coronary artery disease, cardiovascular-related mortality, stroke, and obesity-related cancers [11]. The review also evaluated 24 nutrition intervention studies examining dietary effects on circulating ceramides [11]. Consistent dietary patterns emerged, demonstrating that saturated fat intake, caloric overfeeding, and high total fat consumption were associated with elevated ceramide levels [11]. In contrast, increased intake of polyunsaturated fats was associated with decreased circulating ceramides [11]. Although the authors noted that dietary evidence remains limited, the collective findings indicate that dietary composition can significantly influence ceramide metabolism [11].

From a mechanistic perspective, ceramides have been shown to impair insulin signaling by inhibiting Akt phosphorylation, advancing mitochondrial dysfunction, and increasing inflammatory signaling pathways, all of which contribute to the development of insulin resistance [11]. These mechanisms provide biological plausibility for the observed associations between elevated ceramides and T2DM across diverse study populations [11]. Together, these findings suggest that ceramides may serve as a link between excess saturated fat intake and the progression of metabolic disease [11].

From a clinical standpoint, the consistent relationship among dietary composition, ceramide levels, and insulin resistance supports the role of nutritional modification as a practical strategy to influence ceramide-associated metabolic risk. While ceramide testing is not yet routinely used in clinical practice, dietary interventions emphasizing unsaturated fats, dietary fiber, and plant-based foods align with existing diabetes prevention guidelines and may offer additional metabolic benefits through modulation of ceramide metabolism.

Limitations

Several limitations within the existing literature should be acknowledged. A primary limitation is the lack of standardized definitions for dietary interventions, particularly for Mediterranean-style diets. Across studies, dietary protocols varied widely or lacked detailed reporting of macronutrient distribution and food quantities, limiting comparability and reproducibility. Additionally, many studies relied on observational designs or short-term interventions, which restricts the ability to establish long-term causal relationships between diet, ceramide levels, and diabetes outcomes.

Other limitations include limited stratification by sex, ethnicity, and baseline metabolic status, as well as variability in laboratory methods used to measure ceramide species. The absence of standardized assays and clinically validated threshold values further constrains the translation of ceramide testing into routine practice. Future research should prioritize standardized dietary protocols, longer randomized controlled trials, and harmonized ceramide measurement techniques to better define clinical utility.

## Conclusions

Current evidence supports plasma ceramides as a promising biomarker associated with insulin resistance and type 2 diabetes mellitus. Elevated ceramide concentrations are consistently observed in individuals with impaired glucose metabolism and are strongly associated with increased diabetes risk. Dietary patterns characterized by excessive caloric intake, high total fat consumption, and elevated saturated fat intake are associated with increased ceramide levels, whereas diets emphasizing unsaturated fats, dietary fiber, and plant-based foods appear to be associated with lower circulating ceramides.

Although ceramide testing shows potential as a complementary metabolic risk marker, its integration into standard clinical practice remains limited by the lack of standardized assays, established clinical thresholds, and approved pharmacologic therapies targeting ceramide reduction. Continued research is warranted to clarify population-specific effects, refine measurement techniques, and evaluate the role of dietary and therapeutic interventions in modulating ceramide-associated diabetes risk.
